# “I rarely read the label”: Factors that Influence Thai Consumer Responses to Nutrition Labels

**DOI:** 10.5539/gjhs.v8n1p21

**Published:** 2015-05-15

**Authors:** Wimalin Rimpeekool, Cathy Banwell, Sam-ang Seubsman, Martyn Kirk, Vasoontara Yiengprugsawan, Adrian Sleigh

**Affiliations:** 1 National Centre for Epidemiology & Population Health, Australian National University, Canberra, Australia; 2 School of Human Ecology, Sukhothai Thammathirat Open University, Nonthaburi, Thailand

**Keywords:** Thailand, nutrition label, qualitative study, consumer, behaviour, community health

## Abstract

**Background:**

This qualitative study employed the Knowledge-Attitude-Behaviour (KAB) model and Health Belief Model (HBM) to investigate factors influencing Thai consumer decision making about use of nutrition labels. Labels include both Nutrition Information Panels (1998-) and Guideline Daily Amounts labels (2011-).

**Method:**

In-depth interviews were conducted with 34 participants representing two socio-demographic extremes in Thailand – “urban Bangkok” (university educated consumers) and “provincial Ranong” (non-university educated consumers). An integrated KAB-HBM model was used to devise in-depth interviews for a qualitative study using 20 open-ended questions and samples of food package labels. Additional questions arose from the interviews and they lasted 30-45 minutes and were video recorded. The analysis identified recurring themes using Atlas.ti software.

**Results:**

Most participants (n=25) were aware of nutrition labels but a much smaller number (n=10) used and derived any benefit from them. Nutrition label users were classified into 4 groups: A) competent user; B) confused user; C) aware non-user; D) unaware non-user. Better educated participants were better at understanding nutrition labels but not more likely to use labels. Belief that nutrition influences health increased likelihood of using nutrition labels to make decisions about food. Being well-educated and motivated by health concerns increased likelihood of attention to nutrition labels.

**Conclusion:**

Results are discussed with a view to increasing the use of nutrition labels by Thai consumers. Our findings, drawing on a combination of the KAB and HBM models, can contribute to strategies motivating consumers to use nutrition labels and can provide useful insights for developing promotional strategies.

## 1. Introduction

A sound understanding of nutrition labels can contribute to healthy food consumption and improve consumer health status (Jasti & Kovacs, 2010; Post, Mainous Iii, Diaz, Matheson, & Everett, 2010; Storcksdieck Genannt Bonsmann & Wills, 2012). In an effort to boost community health, the Thai Ministry of Public Health (MOPH) introduced a Nutrition Information Panel (NIP) in 1998 on specific prepackaged foods. Despite these efforts the prevalence of overweight and diet-related disease has grown among Thai adults as has the national trend towards unhealthy diets. Obesity prevalence in Thai adults (35-59 years old) had steadily increased from 1991 to 2009 (Wibulpolprasert, Sirilak, Ekachampaka, & Wattanamano, 2011). More than four fifths of adults consume insufficient amounts of vegetables and fruit (Institute for Population and Social Research - Mahidol University, 2011). Furthermore, diet-related conditions such as cardiovascular disease will have a significant impact on Thai health into the future (Zhao et al., 2014).

Reacting to concerns about over-nutrition and Non Communicable Diseases (NCDs) the MOPH took further steps by designing a more comprehensible version of the nutrition label (Royal Thai Government Gazette, 2011). The Guideline Daily Amounts (GDA) nutrition label was introduced in 2011 for four categories of risk-related food types–energy, sugar, fat, and sodium. This “Hwan-Man-Khem label” (sugar-fat-salt label) is now compulsory on the front of packages of five groups of popular processed snack foods - fried or baked potato chips, fried or baked popcorn, rice crisps or extruded snack, crackers or biscuits, and filling wafers. Currently, Thai snack foods display both NIP and GDA on their packages.

Despite these efforts, food labelling is a relatively under-researched topic in the Thai context. Since 2009, only two quantitative surveys have been conducted with Thai consumers. The first found 89% of respondents were aware of nutrition labels, 54.4%, understood them and 62.8% used the information on them (Parinyasiri, 2010). One year after GDA labels were added to the five snack foods listed above, the Thai Food and Drug Administration (Thai FDA) reported that an average of 48.1% of respondents were aware of GDA labels, 63.3% understood them, and 52.4% were able to apply information from GDA when choosing products (Yodtheun, Juntarasuthi, Rochanawanitchakarn, Ratanatikaumporn, & Panprayun, 2013). These studies demonstrated that consumers’ awareness and use of labels is comparatively low but they did not address the reasons for this. Little is known about Thai consumers’ decision making processes related to using labels nor their responses when shown real packaged foods.

For labels to be effective, consumers need to be aware of, understand, and make use of them. Our study sought an in-depth understanding of Thai consumers’ responses to actual nutrition labels on existing food products so that strategies to improve the use of labels could be developed. It aimed to contribute to better health promotion strategies both in Thailand and in other South East Asian countries that are undergoing a nutrition and health transition related to a rapidly westernizing diet and rising NCDs.

## 2. Methods

### 2.1 Participants

The study was conducted in two areas: urban Bangkok and provincial Ranong. Bangkok residents were recruited by phone from members of the Thai Cohort Study (TCS) (Sleigh, Seubsman, Bain, & the Thai Cohort Study Team, 2008; Seubsman, Yiengprugsawan, Sleigh, & the Thai Cohort Study team, 2012), a large national cohort of students from Sukhothai Thammathirat Open University under study since 2005 for the health-risk transition. Ranong residents were recruited inside the only supermarket (Tesco Lotus) in that province, but only if they did not have a university education. The aim was to include participants with a range of educational and health knowledges in the age-groups likely to benefit from future nutrition labelling (20-45 years).

### 2.2 Interviews

Health-related behaviour has long been studied using conceptual models (Egger, Spark, & Lawson, 1992; Contento, 2010) such as the Knowledge-Attitude-Behaviour (KAB) Model in which knowledge mediates attitudes and behavioural change. Also useful is the Health Belief Model (HBM) which considers vulnerability combined with belief that prevention is possible, leading to action to reduce risk. These two well established models of health behaviour were integrated and adapted to qualitatively explore the influence of nutrition labelling on nutrition-related health behaviour.

The interview protocol was based on the integrated KAB-HBM model of health behaviour and from similar international qualitative research on nutrition labelling (Marietta, Welshimer, & Andersons, 1999; Maubach & Hoek, 2010; Wahlich, Gardner, & McGowan, 2012; McLean & Hoek, 2014). Interviews were conducted face-to-face by the lead author in Thai using a semi-structured schedule of questions. Each participant was given examples of real food packages with both NIP and GDA labels and was asked to explain the information on the labels and their reaction to them. An open-ended approach adapted from grounded theory (Glaser & Strauss, 2009) was used in interviews. Each interview lasted around 30-45 minutes and was audio and video recorded with participants’ consent. The number of interviews conducted was guided by the principle of saturation, or the notion that little new information was being gained in additional interviews. Once this occurred in each setting we refrained from collecting more interviews. This study’s protocol was approved by the Australian National University Human Research Ethics Committee (Protocol 2013/148).

### 2.3 Data Analysis

The audio content was transcribed verbatim into Thai text and cross-referenced with the video recording to help validate the verbal transcript with body language. The transcripts were read repeatedly to determine appropriate code words that reflected the overall research questions and the components of the two models (KAB, HBM) that guided the research as well as new or emergent ideas expressed in the interviews. The code list was approved by team members before the textual data was uploaded to Atlas.ti software which was used to apply codes words to the text and identify reoccurring themes. The results of the preliminary analysis were transcribed into English and discussed with the research team to refine themes before writing up final conclusions. Direct quotations have been edited to improve their readability.

## 3. Results

Equal numbers of male and female participants (n=34) aged between 20 and 45 years were recruited. University educated (UE) Bangkok participants were expected to have been exposed to more nutrition information via advertising than those living in Ranong, non-university educated (NUE) participants. Slightly more Ranong (n=20) than Bangkok participants (n=14) were recruited as some Ranong participants did not know enough about food labels to discuss the topic in detail.

### 3.1 Using Nutrition Labels

Most participants were aware of NIP labels (n=25) but less than half of them (n=10) used the labels and derived any benefit from them. In contrast, 6 participants reported that they have seen GDA, although only one person knew the Thai term “Hwan-Man-Khem label” for GDA, and none were familiar with the English term “GDA”. Finally, we grouped participants by their behavioural responses to NIP (Table 1) to gain a deeper understanding of barriers impacting on the utility of labels.

**Table 1:** Groups of nutrition label users

Group	Users’ characteristics	n
A	Competent users who were aware of nutrition labels, had correctly used them, and received benefit in everyday life	10
B	Confused users who were aware of nutrition labels, had used them, but had difficulty interpreting	9
C	Aware non-users who did not use nutrition labels	6
D	Unaware non-users who had not used nutrition labels nor received any benefit from them	9

Participants in Group A were predominantly university educated (n=8/10) and were aware of, and had correctly used, both types of nutrition labels in their everyday life. They were likely to be concerned about diet and health. For example, a well-educated, middle-aged woman in Group A was influenced enough by the cholesterol content of a pre-packaged food to alter her behaviour.

*In the last few days, I went to purchase frozen food [pasta with Carbonara sauce]. When I read the label, I changed my mind [because it contained high cholesterol]*.

This group also contained participants without a university education who used labels because they were motivated by health concerns.

In contrast, Group B participants were aware of and had used nutrition labels but they had difficulties in interpreting them and applying the information correctly. In the interviews some participants confused the nutrition labels with other food labels or made comments like “I have read it but I did not understand it”. This woman demonstrated confusion, common amongst participants, between ingredient lists and nutrition information. Nutrition labels do not list ingredients but she thought as follows:

It is this ingredient [list] label. … I have read it before. If it displayed flavour enhancer, I will not let my child eat it. (NUE, male, age 34)

Group C participants, which included both university educated and non-university educated participants, were aware of nutrition labels but did not read them for a variety of reasons including that the font is too small, or they did not see any reason for using them. They often stated that “I rarely read the labels”. One university educated man explained:

I never use them. I have seen them but did not think to use them. (UE, male, age 41)

Group D contained participants who were unaware of, had not used, nor received any benefit from nutrition labels. Their reasons for not reading or using labels included that the labels contained “too much information”. They also said things like: “I don’t understand” or they indicated that their food preferences were more important than health consideration. As this person said “I prefer a tasty product”. This 37 year old Ranong man who did not have a university education provided an interrelated set of reasons that included his own level of knowledge and lack of education which contributed to his lack of “attention”:

*I think that I cannot read the label because I don’t understand deeply. … I didn’t study [enough about it]. I looked over it and didn’t pay attention to it*.

### 3.2 Knowledge

#### 3.2.1 Dimensions of Knowledge Affecting Awareness of Nutrition Labels

As we have already illustrated, general levels of education as well as specific nutrition knowledge contribute to participants’ ability to use nutrition labels. All university educated participants were aware of NIP while those without a university education were less likely to be aware of labels. Education level is associated with literacy and numeracy skills, as well as nutrition knowledge. Highly literate participants were able to develop their skills in using nutrition labels through frequent application in everyday life. For example, this university educated woman even demonstrated her familiarity with the history of labels when shown a package during the interview.

*I see that [NIP] is available. I saw it when I turned [package] over and then I read it.… I think it [first] began to appear on dietary supplements, and then they appeared on general foods*.

This highly educated man provides another example of a participant who had learned to use nutrition labels through repeated use.

I know [nutrition labels] by myself. Nobody told me. Just reading and keep reading. (UE, male, age 36)

Generally though, consumers did not engage with labels due to lack of education and nutrition knowledge which affected their understanding of, and confidence in, interpreting and using information on nutrition labels. Non-university educated participants had more difficulty in identifying the nutrition labels on real packages during the interview because they could not understand the information provided. One 32 year old non-university educated woman exemplified the many problems that people have with labels. As she so clearly illustrates, consumers not only need to understand the terminology used they also require an understanding of how label information connects to general health concepts and their own particular health needs.

*I don’t understand really. I don’t understand and I have never learned [about nutrition labels]. I don’t have knowledge regarding what total fat is. What is saturated fat? Cholesterol? Protein? Mineral? Dietary fiber? All this information [on nutrition labels]? I don’t know how they benefit my body. Which is an advantage? Which is a harm? I really don’t know*.

#### 3.2.2 Sources of Nutrition Label Knowledge

Participants had not received any education about nutrition labels at school or university. Instead, they gained some awareness from watching television but it was not enough to develop their knowledge or influence their use of labels as one man explained:

I know [nutrition labels] from advertisements and television media. … I saw them but I did not pay attention. … I saw them and I did not understand. What is percentage? I don’t understand about percentage. (NUE, male, age 40)

However, participants who learned about labels via the internet tended to understand them better. The internet provides more interactivity than television allowing consumers to improve their understanding of labels over time although internet information is not always accurate and can lead to misinformation. Two participants were well-educated and familiar with the internet because they worked with computers.

I got [information about health and eating] from a website. … The [website] said that … information [on NIP] did not show calories per package but it actually showed calories per serving. (UE, female, age 37)

I got it [health and diet knowledge] from the internet. I often search health topics when I have free time. (NUE, female, age 37)

Other sources of information included books, health club memberships, and workshops. Many participants also reported that their workplaces provided annual health check-ups, and workshops on healthy eating but only one mentioned learning about nutrition labels.

I have known about [nutrition labels] from a public health workshop. … My previous office sent me to learn from the public health service. … They might want me to use it for public relation purpose [my career] and for myself.” (UE, female, age 30)

### 3.3 Attitudes and Beliefs: Motivation vs Likelihood of Using Nutrition Labels

Negative results from an annual health checkup and medical diagnosis were often a “turning point” that changed a participant’s attitude to their eating habits, which then led them to consult nutrition labels. Annual health checkups sometimes revealed problems such as high blood cholesterol, high blood sugar, and high blood pressure, which led to a recommendation from a physician to eat healthier diets. Being diagnosed with such a problem encouraged participants to use nutrition labels. For example, this nutrition label user changed his diet due to a high blood cholesterol result.

Three years before, I ate normally. I ate deep-fried food and squid. My turning point was a health check-up. Before this, my age was not too high so I did not mind [about food choice]. I was not a fat guy. After health check-up, I found that my blood cholesterol was too high. (UE, male, age 36)

Some participants also modified their attitudes and eating behaviours without a medical diagnosis when they believed that they were at risk of a diet-related health issue such as increasing age, weight gain, changing appearance, and slow body metabolism.

Recently, I begin to control my weight. After I got pregnant, my weight did not decrease even though I ate the same. … For me, it is important because if I get diabetes, many bad effects will come to me. (UE, female, age 29, BMI 24.84)

I look for sodium. Have I got too much [sodium]? Sometime I wonder if I’ve got disease [hypertension]… I try to find the cause of symptoms by reading labels and to cut out something bad for me. (NUE, female, age 37)

Other people’s experiences or a family member’s illness also motivated participants to change their eating behaviour. These examples show that some participants had quite high levels of awareness of diet-related health risks.

I enjoyed eating and never mind about sodium. … After my friend [who graduated in nutrition] told me, I have begun to look at [nutrition] labels. (UE, female, age 29)

Because I am afraid of illness and obesity. … They [public health service mobile unit] came to set up a booth. They have a blood check-up service, blood pressure measurement, and also giving hospital brochure. … Many food sellers went to get a check-up. Most of them have diabetes and high blood pressure. (NUE, male, age 40)

### 3.4 Behaviours: Consumers Choose not to Read Nutrition Labels

Some participants, like this well-educated woman did not see any need to read nutrition labels in general.

I rarely read [nutrition labels]. I think it depends on my interest. I have seen advertisements about recommended intakes of sugar and salt. I know all of that but I did not much mind it. (UE, female, 40)

This man acknowledged that nutrition labels are useful but he did not want to use them because he did not have a severe health problem.

Now, I just know it is really useful. It is useful for a person who is unhealthy. It is a way for controlling [health status]. (NUE, male, age 37)

Once participants were familiar with a food they no longer read the label which meant that they were unaware if a product changed its nutrient content.

If it is a new product, I will read the food labels only the first time [purchasing] because they do not change anything. (UE, male, age 41)

Other considerations were sometimes more important. This young non-university educated woman said “I read only the price tag and my favourite flavour”. Another woman reported that she mostly bought foods that she liked saying; “I had tasted a flavour and [it was] delicious, I never read the nutrition labels”

## 4. Discussion

Overall, more participants were aware of NIP (n=25/34) than GDA (n=6/34) indicating that the placement of food labels is important. Those with higher levels of education or with a relevant health condition were more likely to use nutrition labels. People’s low levels of literacy, numeracy and nutrition knowledge often limited their ability to correctly use nutrition labels due to complexity of information provided. Some participants chose not to use them because they did not have health problems or they put other considerations first.

Thus far, only two national studies of nutrition label use by Thai consumers have been conducted (Parinyasiri, 2010; Yodtheun, Juntarasuthi, Rochanawanitchakarn, Ratanatikaumporn, & Panprayun, 2013). In one study, the Thai FDA reported that 62.8% of respondents in their survey used NIP (Parinyasiri, 2010). This study reported 54.4% of respondents understood NIP but a later Nielsen global study found that only 27% of Thai consumers understand most information on NIP (Nielsen Consumer Research, 2012). However, it should be noted that these differences could be due the different wording used in the questionnaires. The proportion of Thai consumers who used NIP was higher than in Malaysia (46.4%) (Norazmir, Norazlanshah, Naqieyah, & Anuar, 2012) and the USA (61.6%) in 2010 (Ollberding, Wolf, & Contento, 2010).

In the only published study of Thai consumers’ responses to GDA, it was found that 52.4% of respondents were able to apply information from GDA when choosing food products (Yodtheun, Juntarasuthi, Rochanawanitchakarn, Ratanatikaumporn, & Panprayun, 2013) Respondents were not asked if they actually used GDA which suggests that usage may be lower than this figure implies. Nevertheless, it appears that Thai use of GDA may be higher than elsewhere. The proportions of the population who actually use GDA across 6 European countries were 16.8%: with UK respondents most likely to use it (27%) and French respondents least likely to (8.8%) (Klaus, Laura, Josephine, Stefan Storcksdieck Genannt, & Liliya, 2010). Once again these differences may reflect different survey approaches.

However, these earlier Thai studies offer only limited insights into the reasons why consumers are not readily adopting nutrition labels. Our use of in-depth interviews has allowed us to investigate consumers’ attitudes and practices more deeply than the usual design employed in other studies (Misra, 2007; Wiles, Paterson, & Meaker, 2009). The Malaysian survey asked about reasons for not using nutrition labels and showed 32.4% reported they do not understand terms on the package (Norazmir, Norazlanshah, Naqieyah, & Anuar, 2012). The use of closed questions did not give consumers an opportunity to explain their attitudes in depth and it did not reveal that there are consumers, like our group B participants, who misunderstood or misused labels. Our approach has revealed that some people had positive attitudes to nutrition labels and commonly read them but incorrectly applied this information to their food choices. We found that consumers were often confused when shown real food labels and that others just did not want to use them even though they were knowledgeable about them.

**Figure 1:**
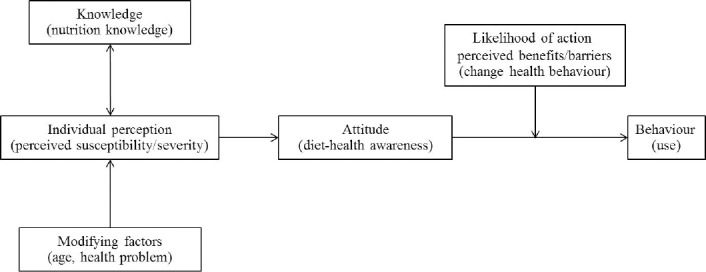
Knowledge-Attitude-Behaviour and Health Belief mixed model (KAB-HBM)

Combining the KAB and HBM models to analyze consumers’ actions reveals that use of nutrition labels requires both adequate knowledge and a corresponding belief in healthy eating. Attitudes are often motivated by a personal health issue, as explained by HBM. In line with another study (Guthrie, Fox, Cleveland, & Welsh, 1995) we found that consumers were more likely to use nutrition labels when they perceived a personal susceptibility to a diet-health problem. We therefore propose that this mixed model (Figure 1) better explains consumer responses to nutrition labels and that this understanding will contribute to more effective promotional strategies.

Most of the existing qualitative studies of nutrition labels have investigated consumers’ understanding and interpretation of label information but few have explored their motivation. One study, with similar results to ours found that participants’ nutrition label use and food choices were related to concerns about specific diet-related disease, general health, and physical appearance (Wahlich, Gardner, & McGowan, 2012). Our study provides additional understanding of the level of health concern which affected the likelihood of people using labels. Participants who received a diagnosis of a serious health condition from their annual health checkup began to use labels and improve their diets. Perceived susceptibility to a diet-health problem or an experience of other peoples’ illnesses also encouraged label use.

Our results suggest that teaching consumers, such as the label-aware non-users (Group C), to interpret nutrition labels without changing their perceptions about eating and health concerns will not motivate them to use nutrition labels regularly. Therefore media and health promotion advertisements should also focus on changing consumer attitudes and on awakening consumer awareness of the connections between health and diet. Sources of information about interpreting nutrition labels may be provided in pamphlets, books, and also in workshops and existing sources of health information. Another avenue would be to disseminate additional nutrition and label knowledge to people who are already concerned with diet and health through groups such as those run in hospitals or special clubs for people with diabetes, hypertension, and metabolic syndrome. We also found that the location and size of GDA labels do not attract consumer attention. So in the short term advertisements (on TV or internet) about GDA should indicate where the label is located to improve consumer awareness. Over the longer term, consideration should be given to improving the position of labels to make them more prominent.

This type of research is not intended to be directly generalizable to the entire community; instead it provides an in-depth understanding of people’s perceptions and practices in Thailand, which may be more widely applicable in South East Asia or other regions undergoing a rapid increase in processed, packaged food consumption. We have documented participant responses to labels on snack foods because they are the only Thai food products that are required by law to have NIP and GDP labels. However, participants’ experiences with nutrition labels may also come from other foods and they may react differently to labels on more essential foods.

This study contributes to existing research on label use in Thailand by providing deeper insights into people’s attitudes and it helps explain why they behave in particular ways. Categorizing nutrition label users into two groups (users and non-users) as is common in survey research does not reflect the more complex ways people respond to nutrition labels. In addition, our findings reveal that there are consumers who do not understand labels correctly even though they read them and are therefore likely to miss out on associated health benefits or may even suffer negative health consequences. This research can inform the development of future survey research in Thailand that will provide more generalizable results and the development of health promotion strategies about labels for general consumers.

## 5. Conclusions

The level of education among participants informed their use of nutrition labels. Also, awareness of GDA was very low. Only one-third of participants actually benefited from nutrition labels. Our results indicate that Thai consumers need additional motivation and encouragement to make better use of nutrition labels. Different groups of label users would benefit from programs or interventions that targeted their level of awareness or knowledge. Consumers’ attitudes are the key to improving their likelihood of using nutrition labels. To promote nutrition label use in the general public, the combined KAB and HBM models could be used to design short messages and heart-warming stories to motivate consumers.
